# Crop Wild Relatives (CWRs) Threatened and Endemic to Italy: Urgent Actions for Protection and Use

**DOI:** 10.3390/biology11020193

**Published:** 2022-01-26

**Authors:** Enrico Vito Perrino, Robert Philipp Wagensommer

**Affiliations:** 1 CIHEAM, Mediterranean Agronomic Institute of Bari, Via Ceglie 9, 70010 Valenzano, Italy; 2 Department of Biology, University of Bari Aldo Moro, Via Orabona 4, 70125 Bari, Italy; robert.wagensommer@uniba.it

**Keywords:** endemism, gene pool, geographic distribution, threats, valorization

## Abstract

**Simple Summary:**

Crop wild relatives (CWRs) are wild plants with an indirect use derived from their relatively close genetic relationship to a crop and have contributed to crop domestication for millennia. Nowadays, due to human overexploitation of plants and other environmental resources, they are threatened and hence need protection to guarantee plant evolution and food supply for the future of human generations. This concept is especially true for endemic CWRs, which have a greater risk of genetic erosion and/or extinction and need more urgent, specific targeted actions. In this study, the Italian threatened and endemic CWRs, often exclusive of one administrative region, are discussed based on the Annex I Priority crops of the International Treaty on Plant Genetic Resources for Food and Agriculture (ITPGRFA, FAO).

**Abstract:**

An updated overview of the 29 threatened crop wild relatives (CWRs) endemic to Italy is presented, namely: *Arrhenatherum elatius* subsp. *nebrodense*, *Barbarea rupicola*, *Brassica baldensis*, *Brassica glabrescens*, *Brassica macrocarpa*, *Brassica rupestris* subsp. *hispida*, *Brassica rupestris* subsp. *rupestris*, *Brassica tardarae*, *Brassica* *trichocarpa*, *Brassica tyrrhena*, *Brassica villosa* subsp. *bivonana*, *Brassica villosa* subsp. *brevisiliqua*, *Brassica villosa* subsp. *drepanensis*, *Brassica villosa* subsp. *tineoi*, *Brassica villosa* subsp. *villosa*, *Daucus broteroi*, *Daucus carota* subsp. *rupestris*, *Daucus nebrodensis*, *Diplotaxis scaposa*, *Festuca centroapenninica*, *Lathyrus apenninus*, *Lathyrus odoratus*, *Malus crescimannoi*, *Phalaris arundinacea* subsp. *rotgesii*, *Vicia brulloi*, *Vicia consentina*, *Vicia giacominiana*, *Vicia ochroleuca* subsp. *ochroleuca*, *Vicia tenuifolia* subsp. *elegans*. Data concerning geographical distribution, ecology (including plant communities and habitats of the Directive 92/43/EEC), genetics (chromosome number, breeding system, and/or the existence of gene pools), threat status at the national and international level (Red Lists), key plant properties, and in situ and ex situ conservation were analyzed and shown. At present, most of the listed endemic CWRs, 23 out of 29, have no gene pool at all, so they are CWRs only according to the taxon group and not according to the gene pool concept. In addition, there is a serious lack of data on the ex situ conservation in gene banks, with 16 species identified as high priority (HP) while 22 taxa have high priority (A) for in situ conservation. With the aim of their protection, conservation, and valorization, specific and urgent actions are recommended.

## 1. Introduction

Crop wild relatives (CWRs) are wild plants with a more or less close genetic relationship to a crop. They have contributed to crop domestication for millennia. The concepts related to the conservation and enhancement of CWRs have been stated in several studies [1,2,3,4,5,6,7]. The FAO has developed the Voluntary Guidelines for the Conservation and Sustainable Use of Crop Wild Relatives and Wild Food Plants, well resumed in the foreword of Ren Wang: “*Crop wild relatives (CWR) thrive in their natural habitats without human intervention. In fact, they are continuously evolving adaptive characteristics that enable them to cope with changing environmental conditions. Therefore, they are a rich reservoir of novel traits and genes that can be used to develop crop varieties that are adapted to climate change. There is ample evidence of their successful use in crop improvement. Wild food plants, on the other hand, constitute important components of the diets of many people across the globe. Though undomesticated, they are rich sources of very important micronutrients, which, sadly, are lacking in the main staple crops that people are increasingly relying on for nourishment. Wild food plants could therefore play critically important roles in combatting malnutrition. Crop wild relatives and wild food plants share one thing in common: their habitats. These natural wild habitats are increasingly under threat from both human activities and natural disasters, implying that the diversity of both crop wild relatives and wild food plants are being continuously eroded. In fact, many could become extinct if the current level of neglect is not checked*” [8], and we could add: not monitored, evaluated, and conserved.

The Italian national checklist of CWRs and WHPs (Wild Harvested Plants) [http://vnr.unipg.it/PGRSecure, accessed on 16 September 2021] identify 11.710 taxa, a very high number, of which 92% (10,773) are CWR/WHP, among which 10–11% (1118) taxa were prioritized, of which 129 with the highest priority [1], that could be preserved with the available national financial resources and expertise.

The CWR and WHP taxa must, therefore, be prioritized as a means of selecting taxa for which active conservation (in situ and ex situ) should start immediately and taxa for which conservation actions can be delayed [8].

Conservation prioritization depends on a number of factors, including the number of CWRs and WHPs in the country, the financial resources available for their conservation, the different needs of the target areas and local communities, as well as the policies and interests of the implementing body.

The rare and threatened CWR species, with disjoint distribution, of phytogeographic or conservation interest, often characterized by populations of few individuals, and therefore listed in the international conventions and/or in the national or international Red Lists, are taxa needing greater attention, as already highlighted for Italy by some authors [1], and for which specific actions have been proposed for their enhancement and conservation [9].

Among these species, there are those with a very restricted distribution range, called “endemic species” [10]. Geographically restricted species are potentially more adversely affected by localized threats. Thus, for species with restricted distribution, the loss of any single population or group of populations may affect the entire viability of the species. Taxa that are known to be endemic to a country or those that occur in only a few countries or regions may be considered vulnerable. Species with a restricted distribution should therefore be given higher priority than species occurring more widely [8,11,12].

This vulnerability becomes even more critical if we consider endemic CWRs with a comparable gene pool to the related cultivated taxon, with which they can exchange genes. Unfortunately, as stressed several times [2,6], not all wild relatives are equally capable of cross-breeding because they have different abilities to exchange genes, which explains the current existence of three different gene pool groups (GP1, GP2, GP3) [13]. The primary gene pool (GP1) includes species that can be directly crossed with the cultivated species to produce fertile breeds. For example, it is easier for *Beta macrocarpa* Guss. (GP1) to interbreed with cultivated chard (*Beta vulgaris* L.), as they have a stronger genetic affinity than other species that are less related, and for that, they belong to more distant gene pools (GP2 or GP3) as in *Brassica macrocarpa* Guss. which has a GP2 relative to *B. oleracea* L. and a GP3 relative to *B. rapa* L.

In Italy, recent studies on CWRs were published by Landucci et al. [1], Magrini et al. [14], Perrino and Perrino [6], Perrino and Wagensommer [9]. Starting from these papers, the present work aims to assess the state-of-the-art of 29 threatened CWRs endemic to Italy, focusing on their distribution, ecology, and in situ and ex situ conservation to draw up the planned actions for their conservation and enhancement. It should be noted that *Thinopyrum corsicum* (Hack.) Banfi (=*Elytrigia corsica* (Hack.) Holub) is not evaluated, as it is endemic to Corsica and reported in the past, by mistake, in Sardinia by some authors [14,15,16]. *Brassica rupestris* Raf. s.l. and *B. villosa* Biv. s.l., both reported in previous works on Italian CWRs [1,6] are discussed at the subspecies level (two for *B. rupestris* and five for *B. villosa*).

A secondary but no less important aim of the present work is to provide information that can satisfy the needs expressed by the EU-white papers on the future science for European policies [https://www.esfri.eu/sites/default/files/White_paper_ESFRI-final, accessed on 26 December 2021]. Thus, we provide information that can be used by policymakers and institutions to move from potentiality to biological conservation strategy.

## 2. Materials and Methods

The study was planned starting from the taxa listed in 2015 by the *Italian Network of Seed-Banks* (RIBES) [http://www.reteribes.it/, accessed on 20 October 2021], representing the update of the census of the *Italian National Institute for Environmental Protection and Research* (ISPRA) [1], based on Annex I Priority crops of the *International Treaty on Plant Genetic Resources for Food and Agriculture* (ITPGRFA) [17], selecting the endemic taxa from those 43 Italian threatened CWRs [6,14], according to the taxon group concept of CWR [2] and not to the gene pool concept [13].

Overall, 29 threatened Italian endemic [18] CWRs were identified, including three species recently described [19,20,21], and therefore not included in the RIBES list, but were evaluated to test new species in view of future work. According to Peruzzi et al. [22], for Italian endemic taxa, we refer to taxa occurring only in Italy, or in Italy and Corsica (France), or Italy and Malta.

The nomenclature of the taxa follows “An updated checklist of the vascular flora native to Italy” [18], while the syntaxonomic references were conceived by several contributions [23,24,25].

The endemic taxa are those reported in Annex I of the International Treaty on Plant Genetic Resources for Food and Agriculture (ITPGRFA) at genus level [17] and mentioned by the Italian Institute of Statistics (ISTAT) for cultivated areas and yield between 2019 and 2021 [26], included in the national and international Red Lists, both at Italian and European level [27,28,29,30,31,32], or in other specific works [19,20,21], taxa reported by the Bern Convention [33], and by the Annexes to the 92/43/EEC Directive [34] (Table 1).

Thus, the following taxa were investigated (Table 1, Table 2 and Table 3): *Arrhenatherum elatius* (L.) P. Beauv. Ex J. Presl & C. Presl subsp. *nebrodense* (Brullo, Miniss. & Spamp.) Giardina & Raimondo*, Barbarea rupicola* Moris, *Brassica baldensis* (Prosser & Bertolli) Prosser & Bertolli, *Brassica glabrescens* Poldini, *Brassica macrocarpa* Guss.*, Brassica rupestris* Raf. Subsp. *hispida* Raimondo & Mazzola, *Brassica rupestris* Raf. Subsp. *rupestris*, *Brassica tardarae* Ilardi, Geraci & Troia, *Brassica trichocarpa* C. Brullo, Brullo, Giusso, Ilardi, *Brassica tyrrhena* Giotta, Piccitto & Arrigoni, *Brassica villosa* Biv. Subsp. *bivonana* (Mazzola & Raimondo) Raimondo & Mazzola, *Brassica villosa* Biv. Subsp. *brevisiliqua* (Raimondo & Mazzola) Raimondo & Geraci, *Brassica villosa* Biv. Subsp. *drepanensis* (Caruel) Raimondo & Mazzola, *Brassica villosa* Biv. Subsp. *tineoi* (Lojac.) Raimondo & Mazzola, *Brassica villosa* Biv. Subsp. *villosa*, *Daucus broteroi* Ten., *Daucus carota* L. subsp. *rupestris* (Guss.) Heywood, *Daucus nebrodensis* Strobl, *Diplotaxis scaposa* DC., *Festuca centroapenninica* (Markgr.-Dann.) Foggi, F. Conti & Pignatti, *Lathyrus apenninus* F. Conti, *Lathyrus odoratus* L., *Malus crescimannoi* Raimondo, *Phalaris arundinacea* L. subsp. *rotgesii* (Husn.) Kerguélen, *Vicia brulloi* Sciandr., Giusso, Salmeri & Miniss., *Vicia consentina* Spreng., *Vicia giacominiana* Segelb., *Vicia ochroleuca* Ten. Subsp. *ochroleuca*, *Vicia tenuifolia* Roth subsp. *elegans* (Guss.) Nyman.

For each wild relative, three levels of attention were considered both for ex situ and in situ conservation (Table 2).

Concerning ex situ conservation:

- *high priority* (HP) for taxa present in the Italian RIBES (*Italian Network of Seed-Banks*) [14] with zero accessions;
- *normal priority* (NP) for taxa present with less than five accessions (from 1 to 4);
- *zero priority* (ZP) for those species present with five or more accessions (from 5 to 140).

Concerning in situ conservation:

- *high level* (A) for the native taxa related to a crop of worldwide and national importance for food and agriculture, which are included in National and/or European Red Lists, and/or International Conventions, and that need specific monitoring/protection measures;
- *medium level* (B) for the native taxa related to important crops, which are not included or are reported as Least concern (LC) or as Data deficient (DD) in the lists mentioned above. However, due to their restricted distribution, they need monitoring. In addition, for most of them, there is no information about their threatened status at the national and local levels. They could be under severe threat in part of their distribution range; therefore, their genetic diversity could also be in need of protection;
- *low level* (C) has not been assessed because it includes non-endemic taxa [1,6].

For a better evaluation of in situ and ex situ conservation, data on vegetation and Habitat of the 92/43/EEC Directive [34] have been included (Table 2).

In addition, the priority was evaluated considering their gene pools (GP1, GP2 and GP3), according to the concept of Harlan and de Wet [13], through the consultation of the checklist www.cwrdiversity.org/checklist/ (accessed on 28 September 2021) [35] and Vincent et al. [36], checking also their in situ and ex situ conservation priorities (Table 3).

The results are shown in alphabetical order by genus and species and discussed individually based on the following aspects:

- geographical distribution;
- reasons of threat and priorities for conservation (both ex situ and in situ);
- ecology, vegetation types (only those recognized from a phytosociological point of view), and/or habitat of Directive 92/43/EEC;
- key plant properties (if available);
- gene pool evaluation;
- expected actions.

## 3. Results

The Italian threatened endemic CWRs, analysed in the present study, are reported in Table 1.

**Figure 1:**
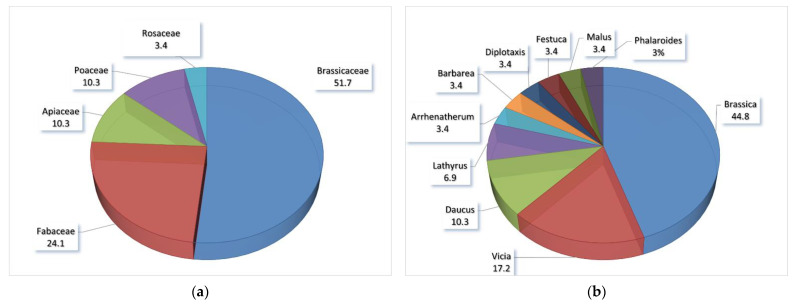
Italian threatened endemic CWR taxa (%) grouped by *family* (**a**) and *genus* (**b**).

According to the taxon group concept, the 29 endemics in Italy belong to the family Brassicaceae (51.7%), with 15 taxa, followed by Fabaceae (24.1%) with 7 species, Apiaceae (10.3%) and Poaceae (10.3%), each with 3 taxa, and finally with only one species by Rosaceae (3.4%) (Figure 1a). The most represented genus is *Brassica* L. (44.8%) with 13 taxa, followed by *Vicia* L. (17.2%) with 5 species, *Daucus* L. (10.3%) and *Lathyrus* L. (6.9%), respectively with three and two species, and finally the genera *Arrhenatherum* P. Beauv., *Barbarea* R. Br., *Diplotaxis* DC., *Festuca* L., *Malus* Mill., and *Phalaroides* Wolf (each with 3.4%)*,* with only one species (Figure 1b).

**Figure 2:**
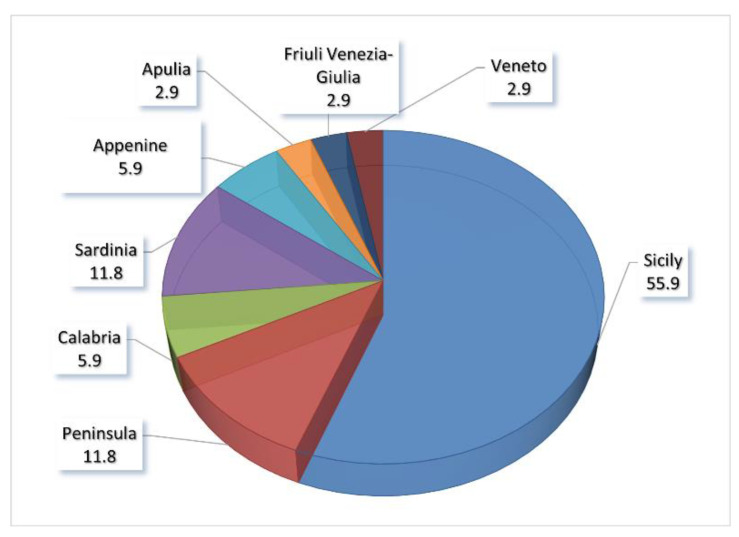
Geographic distribution of Italian threatened endemic CWR taxa (%).

In Italy, these species are not uniformly distributed at the regional level but are more abundant in the central-southern regions, especially in Sicily, which hosts more than half of all evaluated species (Figure 2).

### 3.1. Ex Situ and In Situ Conservation

The results show that for in situ conservation, 22 species have the highest level of priority (A), while the other 7, not included in any National and European Red Lists nor in International Conventions, or reported as Least concern (LC), need monitoring actions (B). Concerning ex situ conservation, 16 species have the highest priority (HP), 5 normal priority (NP), and 8 zero priority (ZP) (Table 2).

#### Relationship between In Situ and Ex Situ Conservation

Globally, many species (22 out of 29) have the highest priority for in situ (A) conservation, while 11 of them, *Arrhenatherum elatius* subsp. *nebrodense*, *Brassica baldensis*, *B. tardarae*, *B. trichocarpa*, *Daucus carota* subsp. *rupestris*, *Lathyrus apenninus*, *L. odoratus*, *Malus crescimannoi*, *Vicia brulloi*, *V. consentina*, and *V. giacominiana* are in the worst situation as they have the highest priority also for ex situ (HP) conservation. For the remaining species, the situation could be considered less hard, as high priority for in situ (A) is balanced by low (NP) or zero (ZP) priority for ex situ (8 taxa with zero priority). No taxon, out of 29, has at the same time zero (ZP) priority for ex situ conservation and medium priority for in situ (B) conservation. In conclusion, many species need monitoring and updating and should be considered at risk, especially those lacking data (Table 2).

### 3.2. The Taxon Group CWR in the Light of the Gene Pool Concept

Since plant breeders would concentrate on wild relatives that may cross easily with crops, we have checked which of the 29 taxa of wild species belong to the three gene pools, foreseen by the Harlan and de Wet [13] concept. The results (Table 3) show that only 6 species out of the 29 belong to one or two gene pools. In particular, *Daucus carota* subsp. *rupestris* shares only the primary gene pool (GP1), while only five further taxa, *Brassica macrocarpa*, *B. rupestris* subsp. *rupestris*, *B. villosa* subsp. *drepanensis*, *B. villosa* subsp. *villosa*, and *Malus crescimannoi*, share the secondary and tertiary gene pools (GP2 and GP3). It is worthy to note that 23 out of the 29 endemics do not have any gene pool, that is, the chance to exchange genes with their hypothetical domesticated/domesticating plant species.

## 4. Discussion

The geographic distribution of the threatened endemic CWR species in Italy (Figure 2) shows that about half of them grow exclusively in the Sicily region. These data can be justified for the peninsular regions, but we did not find a rational reason for Sardinia, comparable to Sicily for geographical extension, climatic characteristics, and floristic similarities [37]. Perhaps, the genus *Brassica* with 13 endemics, of which 9 are exclusive to Sicily, with 5 subspecies of *B. villosa*, may explain this discrepancy in the data.

It is also true that Sicily has a greater extension of cultivated environments in relation to Sardinia and is one of the main centers of the diversification of wild taxa of the *Brassica* sect. *Brassica* in the Mediterranean basin favors the crossing with the cultivated species [21,38,39].

According to the threat status at the national and international level and the number of accessions in seed-banks (Table 1, Table 2 and Table 3), most of the Italian threatened endemic CWRs need high in situ protection (22 taxa out of 29) and/or high ex situ protection (16 taxa out of 29). For this reason, in-depth studies on the distribution and ecology of these species are indispensable. Furthermore, an investigation is needed on the consistency of the populations and on the ability to ripen the seeds. In the following sections, we provide the necessary and currently available information to improve the in situ and ex situ conservation conditions of these threatened taxa.

### 4.1. Arrhenatherum elatius *(L.) P. Beauv. ex J. Presl & C. Presl subsp.* nebrodense *(Brullo, Miniss. & Spamp.) Giardina & Raimondo*

The genus *Arrhenatherum* Beauv. grows with several species in the Mediterranean area, some of which are at risk and need further investigation [40]. The Sicilian endemic, *A. elatius* subsp. *nebrodense* belongs to the cycle of *A. album* (Vahl) W.D. Clayton [41] shows some morphological relationships with *A. palaestinum* Boiss. from the eastern Mediterranean area. *A. elatius* subsp. *nebrodense* is an orophilous taxon, described for the first time by Brullo et al. [40] in the northern mountains of Sicily (Nebrodi, Madonie, Peloritani, and Sicani) and in Mt. Etna and Hyblean territory, between 900 and 1500 m a.s.l.

Nemoral geophyte is reported in deciduous oak woods, rarely in mountain scrub communities and screes [40]. In the *Quercus cerris* L. woods, it occurs as a characteristic species of *Arrhenathero nebrodensis-Quercetum cerridis* Brullo, Minissale, Signorello & Spampinato 1996 association, the community described in northern Sicily, in a belt between the beech forests and the deciduous thermophilic oak forests, is widespread on the Nebrodi mountains, especially along the northern slopes. It is also considered as a diagnostic taxon in the screes of *Linarion purpureae* Brullo 1984 (habitat 8130: Western Mediterranean and thermophilus scree) (Table 2), Sicilian and southern Italy endemic alliance, a pioneer on calcareous, dolomite, or pyroclastic screes in the montane belt and, more rarely, in the basal belt, floristically rather poor.

This taxon shows a high in situ (A) and ex situ (HP) priority conservation, with still no data about chromosome number and gene pool (Table 2 and Table 3). To be precise, according to Kamari et al. [42], the chromosome number of *A. elatius* is 2*n* = 28, a widespread tetraploid cytotype, as the existence of the diploid cytotype (*n* = 7, 2*n* = 14) is mentioned by three authors only on material from Turkmenistan and Spain [41,43,44,45]. In addition, Mehra and Sharma [46], in a study on Himalayan material, mentioned an octoploid cytotype (*n* = 28). Yet, to our knowledge, no one has reported the chromosome number of *A. elatius* subsp. *nebrodensis*.

#### Expected Actions

- In situ and ex situ conservation to prevent the risk of extinction by increasing the number of individuals of existing wild populations;
- ex situ conservation of wild populations is necessary, especially to avoid species extinction or further genetic erosion after ecological changes, and can be realized by plant conservation in botanical gardens and seed-banks;
- genetic studies to check the chromosome number and to eventually define the existence of a gene pool.

### 4.2. Barbarea rupicola *Moris*

The genus *Barbarea* R. Br. is a wild genus belonging to the tribe *Arabideae* along with *Arabis* L., *Cardamine* L., *Cardaminopsis* Hayek, and *Rorippa* Scop. [47], and is composed of 29 species distributed in the warm regions of Eurasia, Australia, North America, in some South American countries, and in the eastern parts of Africa [48]. In Italy, seven species of *Barbarea* (*B. bracteosa* Guss., *B. intermedia* Boreau, *B. rupicola* Moris, *B. sicula* C. Presl, *B. stricta* Andrz., *B. verna* (Mill.) Asch., and *B. vulgaris* R. Br.) are reported [18], of which *B. rupicola* and *B. sicula* are very rare. *B. rupicola* is a casmophytic taxon endemic to Sardinia and Corsica [49,50], indifferent to lithological soils, growing between 600 and 1200 m a.s.l. [51], which can be stressed if disturbed by grazers [52]. This taxon was reported for several places, often dating to several years ago, in different areas of Sardinia, such as the mountains of “Sette Fratelli” [53,54], “Rio Cannas” basin [55], and “Mt. Arbu” (Cagliari) [51] (South East), Punta Sebera [56] (South Western), Mt. Limbara (North) [57], and in an unspecified site of the Northwestern region sector [58].

The floristic studies mentioned above have not been followed by rocky vegetational studies of hilly and mountain belts.

Species of the genus *Barbarea* are subject to considerable attention in the field of chemical ecology [59] and experimental taxonomy [60]. Unique to the *Brassicales*, some species in the genus *Barbarea* build up resistance against herbivorous insects using glucosinolates, which are used in plant defense [61], and produce triterpenoid saponins that are highly deterrent to some specialist herbivorous insects [62].

This taxon shows a medium in situ (B) and ex situ (NP) priority conservation, but it seems that no data are available about chromosome number and gene pools with any domesticated plant (Table 2 and Table 3).

#### Expected Actions

- Although framed with the Least concern (LC) category (Table 1), the available data suggest “*ad hoc*” studies to verify the real extension of the populations.
- phytosociological studies to evaluate vegetation, habitat, and ecology, for which there is a lack of data, with attention on the load-size grazing;
- genetic studies to discover eventual gene pools;
- identify the chemical profile for its possible introduction in agronomy, as done for other species of the genus *Barbarea* considered as a model for evolution and ecology for the plant biological defense due to its unusual glucosinolate profile and production of saponins.

### 4.3. Brassica *L.*

Many crop species are included in the genus *Brassica* L., which provides edible roots, leaves, stems, buds, flowers, and seeds. Many wild relatives have potential as sources for oil, condiments, and other products [63]. Wild taxa in *B. oleracea* L. aggr. play an important role in improving cultivated crops, but the genomic relationships between wild and cultivated forms have not been well clarified [9,64].

An interesting study conducted on tocopherol, fatty acid, and phytosterol content in the seeds of several endemic wild taxa of Sicilian *Brassica*, suggested that they could be exploited in breeding programmes to develop genotypes with enhanced antioxidant capacities and nutritional value [65].

The genus *Brassica* is one of 51 genera in the tribe *Brassiceae*, belonging to the Brassicaceae family, and is the economically most important genus within this tribe, containing 38 different species [66].

In Italy, 24 taxa of genus *Brassica* are reported [18,21], 13 of which are regional endemisms, 9 endemic to Sicily and 3 to different regions: *B. baldensis* to Veneto, *B. glabrescens* to Friuli-Venezia Giulia, *B. tyrrhena* to Sardinia. Only *B. rupestris* subsp. *rupestris* is reported in two regions: Sicily and Calabria (Table 1). The high genetic diversity levels detected in the Sicilian populations of *Brassica* are probably related to the refugial nature of some of these areas during climatic changes, when these areas could have acted as genetic reservoirs of ancestral variation [67,68].

All taxa show a high in situ (A) priority conservation, except *B. tyrrhena* (B), while within ex situ priority conservation, eight taxa have zero priority (ZP), three high priority (HP), and two normal priority (NP) (Table 2 and Table 3).

The gene pool is known only for 4 out of the 13 endemic taxa considered: *B. macrocarpa*, *B. rupestris* subsp. *rupestris*, *B. villosa* subsp. *drepanensis*, *B. villosa* subsp. *villosa*. In particular, *B. macrocarpa* has a GP2 relative to *B. oleracea* L. and a GP3 relative to *B. rapa* L., while the other three *Brassica* taxa have a GP2 relative only to *B. oleracea* (Table 3). To the best of our knowledge, until today, no GP1 has been recorded.

#### 4.3.1. *B. baldensis* (syn.: *Brassica repanda* (Willd.) DC. subsp. *baldensis* (Prosser & Bertolli) Prosser & Bertolli; *Guenthera repanda* (Willd.) Gómez-Campo subsp. *baldensis* Prosser & Bertolli)

*B. baldensis* is a hemicryptophyte (sometimes chamaephyte) caespitose taxon, whose most consistent populations are located between 250 and 820 m. a.s.l., often in dry, rocky habitats (code 8210), on sunny limestone near Mt. Cimo, a minor elevation of Mt. Baldo, inside Special Area of Conservation (SAC) “Mt Baldo East” [IT3210041] (Veneto) [69], a mountain overlooking Garda Lake. Flowering is from April to early May, rarely in June, while the fruiting is at the end of June or in July. Among the insects on the flowers are *Oxythirea funesta* (Coleoptera), *Cantharis* sp. (Coleoptera), and *Blasticotoma* sp. (Hymenoptera) [determinavit by A. Martinelli in [69]. In the Red List of the Italian endemic species, it is reported as Vulnerable (VU) (Table 1). This taxon shows a high in situ (A) and ex situ (HP) priority conservation, and no data about chromosome number and gene pool are known (Table 2 and Table 3).

##### Expected Actions

- In situ and ex situ conservation actions to prevent the risk of extinction by increasing the number of individuals in existing wild populations;
- ex situ conservation of wild accessions is necessary to avoid further genetic erosion due to ecological changes, to realize by plant and/or seed conservation in botanical gardens or seed banks;
- genetic and breeding studies to discover possible gene pools;
- phytological studies to identify the syntaxon.

#### 4.3.2. *B. glabrescens* Poldini (syn.: *Brassica repanda* (Willd.) DC. subsp. *glabrescens* (Poldini) Gómez-Campo)

*B. glabrescens* is a hemicryptophyte taxon reported in a restricted area, in open environments between Cellina torrent and Meduna river in the municipality of Pordenone (Friuli-Venezia Giulia), flowering and fruiting in the April–May period. Hydrocore dispersion is linked to the intermittency of the waters, or zoocore, through transhumant grazing [70]. The chromosome number is 2*n* = 20 on material coming from the Magredi di San Quirino [71]. The species is characteristic of the *Centaureo dichroanthae-Globularietum cordifoliae* Pignatti 1953 association, of *Saturejion subspicatae* (Horvat 1974) Horvatić 1975 alliance and *Scorzonero villosae-Chrysopogonetalia grylli* Horvatić & Horvat in Horvatić 1963 order, ascribable to the “Eastern sub-mediterranean dry grasslands (*Scorzoneretalia villosae*)” habitat of Directive 92/43/EEC (code 62A0), including these grasslands of north-eastern Italy, known by the local term of “magredi” [72]. This species is very rare in plant communities related to habitat “Alpine rivers and the herbaceous vegetation along their banks” (code 3220), along the banks of alpine rivers [70]. The species is included in the Red Lists, as VU or NT (Table 1), in Annex I of the Convention of Bern, in Annex II of the Directive 92/43/EEC, and finally in Annex I of the Regional Law 9/2007 of the wild flora of community interest, that declares the prohibition of collection. The following potential threats through the IUCN classification (2012) [73] have been identified, without data on population size and conservation status of habitat [70]: *Annual and perennial non-timber crops, Livestock farming and ranching, Recreational activities, War, Civil Unrest and Military Exercises, Dams and Water Management/Use, Other ecosystem modifications, Invasive non-native/alien species Named species, Nutrient loads*. This taxon shows a high in situ (A) and normal ex situ (NP) priority conservation and absence of gene pool (Table 2 and Table 3).

##### Expected Actions

- In situ conservation actions to prevent the risk of extinction by increasing the number of individuals in existing wild populations;
- checking the numerical consistency of the existing populations;
- genetic studies to define any eventual gene pool.

#### 4.3.3. *B. macrocarpa* Guss. (syn.: *Eruca macrocapa* (Guss.) Caruel)

B. macrocarpa is a suffruticose chamaephyte taxon, flowering from January to March, with entomophilous pollination. The seeds ripen in early June, after a short time of dormancy, and have a germination percentage between 80% and 100% [74]. The chromosome number is 2*n* = 2x = 18 [75]. It is an exclusive species of Egadi archipelago at Favignana and Marettimo islets in the province of Trapani, in western Sicily [76,77], with 8 sites, 4 on each islet [78], where the species grows on limestone cliffs and rocky ridges near the sea at an altitude between 0 and 300 m a.s.l. From a phytosociological point of view, the species is a differential element of a peculiar chasmophytic vegetation under the influence of wet marine currents, framed into the *brassicetosum macrocarpae* Brullo & Marcenò 1979 subassociation [79], of the *Scabioso creticae-Centaureetum ucriae* Brullo & Marcenò 1979 association and the *Dianthion rupicolae* Brullo & Marcenò 1979 alliance, which falls within the habitat “Calcareous rocky slopes with chasmophytic vegetation” (code 8210). It is also found as a sporadic element in *Periploco angustifoliae-Euphorbietum dendroidis* Brullo, Di Martino e Marcenò 1977 syntaxon in rocky environments in contact with the *Dianthion rupicolae* community, as well as in the *Euphorbietum dendroidis* Guinochet in Guinochet et Drounieau 1944 subass. *typicum*, considered as habitat 92/43 EEC “*Thermo-Mediterranean and pre-desert scrub*” (code 5330) [80]. The species is included in the Red Lists, always as Critically endangered (CR) and only one time as Endangered (EN), in Annex II of the Directive 92/43/EEC as a priority species, and in Annex I of the Bern Convention (Table 1).

The critical issues to which this species is subjected are grazing, especially goat grazing, which represents a barrier to its spread, and the soil altered by the limestone extraction for the construction of roads. A serious threat can be derived from invasive alien species, which are increasing in Italy [37,81], including *Cenchrus setaceus* (Forssk.) Morrone (syn.: *Pennisetum setaceum* (Forssk.) Chiov.) in expansion, fires, and tourism often cause indiscriminate, massive, and unregulated collection [82]. In situ conservation strategies have been undertaken to protect the habitat to ensure the natural development of the evolutionary processes of populations, and ex situ preservation, in seed banks of germplasm representative of their genetic variability. The two known populations fall within two Special Areas of Conservation (SACs), called “Isola di Marettimo” (code ITA010002) and “Isola di Favignana” (code ITA010004), and in the Special Protection Area (SPA) called “Arcipelago delle Isole Egadi” (code ITA010027), which should guarantee their conservation in the medium and long term. In addition, as ex situ protection measures, 5 accessions of seeds from the population of Favignana are kept in the Germplasm Bank at the Department of Botanical Sciences of the University of Palermo [83], 12 accessions was reported in the European database for *Brassica* (Bras-EDB) [84], and other propagation material is stored at the genebank of Department of Agricoltura, Alimentazione e Ambiente of University of Catania [85].

This taxon shows a high in situ (A) and zero ex situ (ZP) priority conservation and the existence of two gene pools (GP2 and GP3). The high in situ (A) priority conservation is explained by the threats mentioned above, while the zero ex situ (ZP) priority conservation is because of the good number of accessions preserved in gene banks (Table 2 and Table 3).

Some authors evaluated the use in tomato crops for its richness of sinigrin in leaves, a glucosinolate (GLS) compound, that showed biocontrol activities against several pests and diseases via nematotoxic action, for the purpose of sustainable management and reduction of chemicals in tomato cultivation for the fight against root-knot nematodes as *Meloidogyne* spp. [86]. A phytochemical characterization of *B. macrocarpa* reports, as for other Sicilian wild *Brassica*taxa, a higher content of flavonol derivatives with respect to *B. olearea* crops, where gentiobiosides are the most representative phenolic compounds. The most abundant compounds isolated in *B. macrocarpa* are kaempferol feruloyl glucoside and kaempferol glucosides [87].

##### Expected Actions

- In situ monitoring by numerical count of individuals, inside the already known populations, during the flowering time (from January to March), and due to the difficulty in identifying the field sites (even from short distances), it is necessary to carefully monitor (even with the use of binoculars) the stations ecologically suitable for hosting the taxon, [82];
- in situ (*on farm*) experiments in cooperation with local farmers, thanks to its high potential agronomic value and high tolerance to drought, insects, and high content of glucosinolates [88], especially sinigrin [86], extending the experiment to tomato and other crops;
- in situ and ex situ crosses with cultivated *B. oleracea* and *B. rapa*, as it is the only Italian endemic *Brassica* taxon with a GP2 relative of *B. oleracea* and GP3 relative of *B. rapa* (www.cwrdiversity.org/checklist/, accessed on 28 September 2021) [35];
- ecological studies are needed to determine the role of grazing (especially by goats) on population maintenance.

#### 4.3.4. *Brassica rupestris* Raf. subsp. *hispida* Raimondo & Mazzola, *Brassica rupestris* Raf. subsp. *rupestris*

*B. rupestris* is a chasmophyte species that includes two subspecies, subsp. *rupestris* endemic to central-western Sicily and southern Calabria [89,90], and subsp. *hispida* endemic of a restricted area that falls within the range distribution of subsp. *rupestris*, in northwestern Sicily and the southern mountains of Palermo [89].

Their phenology has to be clarified, such as their uncertain reproductive biology [91], because considering subsp. *hispida* it was originally reported with a flowering time from March to May [89], while later for both subspecies (subsp. *hispida* and subsp. *rupestris*), it was reported in a generic way with a flowering time from December to April with fruiting from May to July [90]. Both subspecies grow on vertical limestone cliffs, with the subsp. *rupestris* from sea level to 1100 m a.s.l. [90], and the subsp. *hispida* from 800 to 1300 m a.s.l. [89].

The subsp. *hispida* is morphologically distinguished from subsp. *rupestris* for slight morphological characters, specifically by glaucous and more densely hairy leaves and in general of small size [89], differences not confirmed by molecular markers [91,92].

From a phytosociological point of view, the subspecies *rupestris* is characteristic of *Dianthion rupicolae* Brullo & Marcenò 1979, an alliance found on Mt. Pellegrino in two different contexts, with two associations: *Diantho rupicolae-Helichrysetum panormitani* Gianguzzi 2020 and *Scabioso creticae-Centauretum ucriae* Brullo et Marcenò 1979 [93] (Table 2).

In the European Red List, the species is reported as Near threatened (NT), while in the National Red List, at the national and regional levels for Sicily, it is Endangered (EN) for subsp. *hispida* and Lower risk (LR) for subsp. *rupestris* (Table 1). The identification of new stations allowed a reduction of the threat level to Vulnerable (VU) in subsp. *hispida* and Least concern (LC) in subsp. *rupestris*, as shown in the updated Red List on endemic vascular flora (Table 1).

The high in situ (A) priority conservation for both subspecies is explained by limited distribution area, especially for subsp. *hispida*, fire and overgrazing threats [94], and relative restrictions due to the presence of only Regional Protected Areas (Madonie and Nebrodi Regional Parks), while zero ex situ (ZP) priority conservation is due to the presence of a good number of accessions in gene banks (Table 2 and Table 3). A secondary gene pool (GP2) is known for subsp. *rupestris*, while it is unknown for subsp. *hispida* (Table 3).

Samples of *B. rupestris* subsp. *rupestris* collected in Calabria showed an extreme richness in vitamins, fiber, and bioactive compounds with phytotherapy properties that are independent of physical soil parameters, while the total antioxidant capacity and the synthesis of carotenoids and glucosinolates are dependent on soil chemical and biochemical properties [95].

##### Expected Actions

- Clarify the phenology of the two subspecies;
- ex situ conservation is good, but it could be improved by collecting individuals with rare genetic markers belonging to subsp. *rupestris*, located at Monte Pellegrino, San Calogero, and Gole Tardara [90], and from the two populations of subsp. *hispida* at Mt. Pizzuta and Mt. Kumeta. Sampling according to the phenology of the two subspecies;
- conservation of *B. rupestris* populations in situ is advisable by maintenance and management of the ecosystems, also through the use of ex situ germplasm for those populations with few individuals or even absent from the protected areas. The in situ conservation actions should be backed by periodic monitoring of habitat status and threat prevention;
- in situ and ex situ crosses with cultivated *B. oleracea* should be promoted in collaboration with plant breeders and agroecologists, also to investigate possible gene pools for subsp. *hispida*;
- the slight morphological differences among the two subspecies could be the consequence of a continuous gene flow from wild to cultivated *Brassica* and vice versa, along with strong interactions of environment and climate. This would support the hypothesis that subsp. *hispida* might be the result of hybridization with *Brassica* crops [89]. All suggests morphological and molecular studies;
- phytosociological studies, especially for subsp. *hispida*;
- for both subspecies, almost unknown because of their rarity, but rich in bioactive compounds and antioxidant capacity, some studies [95] suggest the possibility to valorize the taxa significantly in the functional food and/or the pharmacological field.

#### 4.3.5. *Brassica tardarae* Ilardi, Geraci and Troia

*B. tardarae* is a puntiform endemic of south-western Sicily, located at “Gole della Tardara”, a few kilometers north–northwest from the town of Sciacca (province of Agrigento) [21]. This taxon is almost similar to *B. rupestris* and *B. villosa* subsp. *brevisiliqua* regarding the indumentum of the leaves, which are glabrous, or with rare bulbose hairs, but it differs from *B. rupestris* in terms of the very short fruit with an evident dorsal rib, while the petals are smaller than in *B. rupestris* and similar to *B. villosa* subsp. *brevisiliqua* for both size and color. Nevertheless, the studied population differs from *B. villosa* subsp. *brevisiliqua* in terms of the morphology and thickness of the basal leaves, the diameter of the seeds, and the habit of the reproductive plants [21].

The flowering time is late January to early February, with the fruiting in mid-May. This chasmophytic taxon grows on the limestone cliffs of the “Gole della Tardara”, between 100 and 400 m a.s.l., inside *Brassico rupestris-Centauretum saccensis* Bazan, Ilardi & Raimondo 2006 association, where it is one of the characteristic species of *Dianthion rupicolae* Brullo & Marcenò 1979 alliance [96]. His habitat is threatened by few human activities as quarries, and consisting of only one population, it has been assessed as Vulnerable (VU), applying the “D” criterion [21].

This taxon shows a high in situ (A) and ex situ (HP) priority conservation, and furthermore, the chromosome number and gene pools are unknown (Table 2 and Table 3).

##### Expected Actions

- In situ and ex situ conservation to prevent the risk of extinction by increasing the number of individuals of the existing wild population;
- ex situ conservation can be realized involving botanical gardens and seed-banks;
- crosses with cultivated *B. oleracea* to check the existence of gene pools;
- try to include Tardara Gorges in a protected area as Natura 2000 sites, or by expanding the perimeter of the neighboring Natura 2000 site “Complesso Monte Telegrafo and Rocca Ficuzza” (code ITA040006).

#### 4.3.6. *Brassica trichocarpa* C. Brullo, Brullo, Giusso, Ilardi

*B. trichocarpa* is a Sicilian endemic taxon of the *B. oleracea* group, with a punctiform distribution, growing at Monte Puccio near San Martino delle Scale (Palermo municipality), on rocky edges at about 900 m of altitude [19]. It is linked to carbonatic soils affected by strong winds and more or less constant misty regime due to the proximity of the Tyrrhenian Sea. The flowering time is May to early June and fruiting late from June to July. The population of *B. trichocarpa* is represented by few (less than 50) and scattered individuals occurring within the thermo-xeric grasslands dominated by *Ampelodesmos mauritanicus* (Poir.) T. Durand & Schinz, ascribable to *Lygeo sparti-Stipetea tenacissimae* Rivas-Martínez 1978 nom. conserv. propos. Rivas-Martínez, Diaz, Fernández-González class, included in Directive 92/43/EEC as “Thermo-Mediterranean and pre-desert scrub” habitat (code 5330).

This taxon is considered Critically Endangered (CR) [19]. This explains the high in situ (A) and ex situ (HP) priority conservation, with no data about chromosome number and the gene pool (Table 2 and Table 3).

##### Expected Actions

- Check carefully the role of *B. trichocarpa* within the *B. oleracea* group, as there are many conflicting views on the taxonomic treatment of suffruticous wild cabbages;
- in situ and ex situ conservation to prevent the risk of extinction by increasing the number of individuals of the single existing wild population;
- ex situ conservation involving botanical gardens and seed-banks;
- crosses with cultivated *B. oleracea*, to prove the inexistence of gene pools;
- phytosociological studies to identify the syntaxon within the *Lygeo sparti-Stipetea tenacissimae* class.

#### 4.3.7. *Brassica tyrrhena* Giotta, Piccitto & Arrigoni

*B. tyrrhena* is a chamaephyte endemic of central-eastern Sardinia, growing on the coastal cliffs of the “Golfo di Orosei” and the internal vertical walls of the “Supramonte” massif, at altitudes between 16 and 520 m a.s.l. [97]. The flowering occurs from February to April and fruiting from May to July. The s’eds’ dissemination is barochore and, secondly, anemochore [98]. The species is reported mainly into the chasmophytic *Helichryso saxatili-Cephalarietum mediterraneae* Arrigoni et Di Tommaso 1991 association, which can be classified in the endemic alliance of *Centaureo-Micromerion cordatae* Arrigoni and Di Tommaso 1991. This plant community is a habitat of community interest, namely “Calcareous rocky slopes with chasmophytic vegetation” (code 8210). Fortunately, the populations of *B. tyrrhena* fall within the Site of Community Importance (SCI) “Golfo di Orosei” (ITB020014), and they are included within the Important Plant Areas (IPAs). Furthermore, with the exception of the Biddiri Scottai and Margheddie (Dorgali, Nuoro municipality) sites, all the other stations are included within the “Golfo di Orosei e del Gennargentu” National Park.

This taxon shows a medium in situ (B) and ex situ (NP) priority conservation. We could not find chromosome number and gene pool records (Table 2 and Table 3). Seeds accessions are preserved at the Sardinia Gene Bank (BG-SAR) and the Millennium Seed Bank (Royal Botanic Gardens, Kew, London, UK).

##### Expected Actions

- check the role of B. tyrrhena within the B. oleracea group carefully, as there are some doubtful intermediate characters between *B. insularis* Moris, from which it is distinguished mainly by the color of the flowers, and *B. rupestris* subsp. *hispida*, from which it differs essentially in leaf morphology and siliques dimensions;
- monitoring of the wild populations and their habitat, strengthening actions, through the help of ex situ conservation;
- ex situ conservation can be continued involving the same botanical gardens and seed banks;
- crosses with cultivated *B. oleracea* to make sure the absence of gene pool.

#### 4.3.8. *Brassica villosa* Biv. Group

*B. villosa* is a Sicilian endemic species with 5 subspecies, i.e., subsp. *bivonana* (Mazzola & Raimondo) Raimondo & Mazzola, subsp. *brevisiliqua* (Raimondo & Mazzola) Raimondo & Geraci, subsp. *drepanensis* (Caruel) Raimondo & Mazzola, subsp. *tineoi* (Lojac.) Raimondo & Mazzola, and subsp. *villosa*. These taxa differ from other Sicilian species of genus *Brassica* by having hairy or pubescent leaves, with the petiole non-auriculate at the base and long more than 30 cm [89]. About thirty sites were identified, several of which were inside protected areas (Madonie and Nebrodi Regional Parks) [94], with the following distribution: subsp. *bivonana* in many localities of the northwestern area; subsp. *villosa* in limited sites in the south of Palermo and on Monte Sicani [99]; subsp. *tineoi* in the central-southern sector; subsp. *drepanensis* in north-east of Trapani [76] and subsp. *brevisiliqua* in a few stations near S. Vito lo Capo at the north-western limit of Sicily [89].

*B. villosa* group grows on limestone, rarely on sandstone, usually north-facing in shaded positions, from sea level to 1000 m a.s.l., in sites where grazing and fire are the main threats [76]. There are few clear vegetation data on these taxa, and referring to subsp. *drepanensis*, a diagnostic taxon of subass. *Typicum* of *Scabioso-Centauretum ucriae* Brullo & Marcenò 1979 association, confined on some sites of Monte Cofano [79,100,101], and to subsp. *tineoi*, a characteristic taxon of *Brassico tinei-Diplotaxietum crassifoliae* association, chasmophytic coenosis endemic to central Sicily and typical of chalky walls [79,102]. These plant communities are a habitat of community interest “Calcareous rocky slopes with chasmophytic vegetation” (code 8210).

The Botanical Garden of Palerm” pre’erves accessions from different sites of all the subspecies collected from 1994 to 1998 [83], while in a cold greenhouse of the experimental agriculture farm of the University of Catania in autumn of 2008, some plants of *B. villosa*, together with other endemic Sicilian *Brassica* species were sown to characterize and compare them based on descriptors at the reproductive stage [94,103,104].

*B. villosa*, which has an exceptionally dense coverage of trichomes (epidermal cells) [105], that act as a barrier for predators and also provide a “blanket” against dehydration and resistance to many pests, may be crossed with *B. napus* L. and *B. oleracea*, although the latter misses the epidermic cell type present in *B. villosa* [106].

A specific study on the fatty acid composition of seed oil showed that *B. villosa*, together with the CWRs *B. incana* and *B. rupestris*, has the highest erucic acid content (>55% of the total fatty acids) comparable with those found in the cultivated species *B. napus*, *B. oleracea*, and *B. rapa* [107]. In addition, the isozyme analyses conducted on 21 species of genus *Brassica*, including Sicilian ones, showed a high degree of genetic affinity between *B. villosa* and *B. rupestris*, whereas *B. incana* and *B. macrocarpa* were clearly more distinct and differentiated [91].

Phytochemical characterization and antioxidant properties of the taxa were studied, showing a high level of the following flavonol derivatives: Kaempferol-3-O-diglucoside-7-O-diglucoside, kaempferol synapoyl glucoside, and quercetin sinapoyl glucoside [87].

All subspecies have a high in situ (A) and zero ex situ (ZP) priority conservation, and only subsp. *drepanensis* and subsp. *villosa* show a secondary gene pool (GP2), while gene pools are unknown for the other subspecies (Table 2 and Table 3).

##### Expected Actions

- The large variation observed among accessions of cultivated germplasm due also to the gene flow from wild relatives suggests the need for promoting their protection and the establishment of genetic reserves [94];
- check the role of subspecies within the *B. villosa* group, with the help of fresh and herbarium material, since the morphological characters seem feeble and eventually convert subspecies to ecotypes. A proof is the likely presence of introgressive hybridization between subsp. *brevisiliqua* and subsp. *drepanensis* [89];
- monitoring populations and their habitat, especially of subsp. *brevisiliqua*, subsp. *drepanensis*, and subsp. *villosa* due to their limited distribution;
- ex situ conservation should be a constant task of botanical gardens and seed banks;
- crosses with cultivated *B. oleracea* and *B. napus*, to establish: (a) the absence/presence of a gene pool in subsp. *bivonana*, subsp. *brevisiliqua*, and subsp. *tineoi*; (b) accepting the trichomes type as a trait for reducing dehydration, select variants more resistant to pathogens and drought;
- phytosociological studies for subspecies poor in vegetation data to evaluate the best strategies for their management and conservation.

### 4.4. Daucus broteroi *Ten.,* D. carota *L. subsp.* rupestris *(Guss.) Heywood,* D. nebrodensis *Strobl*

The genus *Daucus* L. is a member of the Apiaceae family, and most species are reported in Africa, Europe, West Asia, and North America and are used with economically important food products [108]. The cultivated carrot (*Daucus carota* L. subsp. *sativus* (Hoffm.) Arcang.) in terms of economy and nutrition is considered the second most popular vegetable in the world after potato [109]. *D. carota* is the most widespread species of the genus *Daucus*, occurring in temperate regions all over the world. A certain degree of gene flow between cultivated and wild *D. carota* has been recorded, causing genetic contamination both of the cultivated and the wild material. The following scientific statements make the concept even more clear: “*Wild materials can provide sources of resistance to a range of biotic and abiotic stresses, providing additional quality to organic and conventional farming. Breeding carrots for higher productivity and quality in all farming systems and greater adaptation of carrot to warmer climates with concentrated human populations will also benefit from diverse collections of well-characterized germplasm.*” [110]. In any case, the need for a thorough evaluation and valorization of *Daucus* genetic resources is urgent, as they can provide a basis for future developments in carrot breeding [110].

In Italy, this genus represents a critical taxonomic group with 17 species (species or subspecies), 3 of which are endemic to Italy [18]. *D. carota* subsp. *rupestris* is exclusive to Sicily and Malta, *D. nebrodensis* of Sicily, while *D. broteroi* is widespread in central-south regions, except Sardinia and Sicily, and is no longer recorded in Abruzzo and Liguria [18,111].

A taxonomic and nomenclatural study on the *D. guttatus* Sm. complex would suggest for *D. broteroi*, previously *D. broteri* Ten. or *D. michelii* Caruel, due to flower and fruit specific diagnostic morphological characters, has a restricted distribution in central and northern Italy [112]. However, there is some doubt whether the species is endemic to Italy, as it is also reported from Turkey [113] and Iran [114], and has often been confused with *D. muricatus* L., which is a taxon with a wider distribution. In addition, *D. broteroi,* though reported in several Italian regions, is always poorly documented, justifying its European IUCN status: Data Deficient (DD) (Table 1). The most recent reports, since 2010, based on herbarium samples, come from Tuscany (with several sites), Liguria, and Calabria, while the data from the other Italian regions are dated prior to 1950 [115]. Recently it has been reported in wetland vegetation of Calabria, inside *Holoschoenetum vulgaris* Br.-Bl. ex Tchou 1948 [116], even if it is not diagnostic of any vegetation type. The taxon is considered with medium in situ (B) and high ex situ (HP) priority conservation, and there are no data about the gene pool (Table 2 and Table 3).

*D. carota* subsp. *rupestris* is an endemic of Malta and Lampedusa [117,118] that grows on the rocky ridges that rise above the high sea cliffs exposed to sea aerosol, and it is a diagnostic taxon of *Erico multiflorae-Coronilletum glaucae* Brullo S., Brullo C., Cambria et Giusso del Galdo association [119], habitat of Directive 92/43/EEC “West Mediterranean clifftop phryganas (*Astragalo-Plantaginetum subulatae*)” (code 5410). In the European Red List, it is reported as Least concern (LC), while in the National Red Lists, at the national level and regional level for Sicily, as Endangered (EN) (Table 1), showing a high in situ (A) and ex situ (HP) priority conservation (Table 2 and Table 3). It is one of the three Italian CWRs, with *Beta macrocarpa* Guss. and *Crambe hispanica* L. subsp. *hispanica*, but it is the only endemic, with a primary gene pool (GP1) [6] (Table 3). The wild *D. carota* subsp. *rupestris* can naturally exchange genes with *D. carota* subsp. *sativus* (Hoffm.) Arcang.

*D. nebrodensis* is a member of the *D. carota* group, often placed in synonymy with *D. carota* subsp. *hispanicus* (Gouan) Thell., *D. carota* subsp. *hispidus* (Arcang.) Heywood [15], or with *D. hispanicus* Gouan [120]. Accordingly, the evaluation of *D. nebrodensis* as an exclusive endemic species of Sicily is made very critical considering the synonymy with *D. carota* subsp. *hispanicus*, reported at the Balearic Sea and western Gulf of Lion coasts, including the northeastern Iberian Peninsula and nearby areas in southern France, central-eastern Iberian Peninsula, all the Balearic Islands, and the Columbretes Islands [120]. In Italy, it grows in a restricted mountain area of Sicily, from 1600 to 1800 m a.s.l., inside perennial grasslands [121]. In the Italian Red List, it is reported as Least concern (LC) (Table 1), showing a medium in situ (B) and high ex situ (HP) priority conservation, and no data about gene pool are available (Table 2 and Table 3) unless we consider valid the synonymy with *D. carota* subsp. *hispanicus*, in that case, it would be a GP1 relative of *D. carota* subsp. *sativus* (Hoffm.) Arcang.

It is worthy of remembering that the chemical composition of Algerian *D. carota* subsp. *hispanicus* showed sixty-eight essential oils, with strongly fungicidal and inhibitory to aflatoxin production [122].

#### Expected Actions

- Test the genuine endemism of *D. broteroi* and D. *nebrodensis*, already recorded in other countries, and often confused with other similar taxa;
- in situ investigation of *D. broteroi* to define its distribution at the regional level, since it is reported as data deficient (DD) in the European Red List;
- crosses with cultivated *D. carota* subsp. *sativus* to obtain varieties with better characteristics from *D. carota* subsp. *rupestris*, and try to investigate any gene pool both for *D. broteroi* and D. *nebrodensis*;
- in situ and ex situ conservation strategies to avoid the risk of extinction of the wild populations of all three *Daucus* taxa, mainly for *D. carota* subsp. *rupestris*;
- phytosociological studies, mainly for *D. nebrodensis*;
- test the chemical composition of essential oils for their potential use in organic farming as a fungicide.

### 4.5. Diplotaxis scaposa *DC.*

The genus *Diplotaxis* DC. (Brassicaceae) currently includes 34 species, plus 14 infraspecific taxa, native to Europe, the Mediterranean basin, SW Asia (up to the Himalayas), and the Macaronesian region, showing a considerable degree of heterogeneity in morphology, genetic molecular markers, chromosome numbers and geographical amplitude [123,124]. The relative closeness to genus *Brassica* L. makes this genus interesting for the potential contribution of useful traits to cultivated *Brassica* species [124].

There is a great interest in some of its species, especially for *D. tenuifolia* (L.) DC. and *D. muralis* (L.) DC., which are gathered or cultivated for human consumption thanks to the pungent taste of their leaves and appreciated in traditional diets of Mediterranean populations where they become a “gourmet food” [125,126], whereas others, which are always edible, such as *D. erucoides* (L.) DC., are frequent weeds in many European perennial crops, such as vineyards [127] and olive orchards [128].

The quantitative and qualitative characterization of glucosinolates produced by different *Diplotaxis* species has recently called the attention of different disciplines as some of these compounds have proved to be active in clinical tests, and in particular for *D. tenuifolia* thanks to high nutritional and antioxidant components, the potential benefit for prevention in cardiovascular and carcinogenic diseases [129]. Furthermore, *D. tenuifolia* (wild rocket), only recently taken into cultivation, is a good example for early domestication processes, from a camp follower to a crop [130,131,132].

Germplasm collections of taxa belonging to this genus are not sufficiently representative and refer mostly to the species used by man as food [124].

In Italy, six *Diplotaxis* taxa are reported: *D. erucoides* subsp. *erucoides*, *D. harra* (Forssk.) Boiss. subsp. *crassifolia* (Raf.) Maire, *D. muralis*, *D. scaposa*, *D. tenuifolia*, and *D. viminea* (L.) DC. Only *D. scaposa* is endemic to Italy [18], specifically Lampedusa island (Municipality of Agrigento, Sicily). This species has a chromosome number 2n = 18 [133]. It is a diagnostic taxon with other species, as *Daucus carota* L. subsp. *drepanensis* (Tod. ex Lojac.) Heywood (=*Daucus lopadusanus* Tineo), of *Filagini-Daucetum lopadusani* Brullo 1985 association, which identifies a peculiar aspect of the ephemeral coastal meadows that are found in the small depressions of the limestone rock with a thin layer of soil [134]. This annual vegetation is an endemic syntaxon of *Stipo-Bupleuretalia semicomposti* Brullo in Brullo, Scelsi & Spampinato 2001 order, and a priority habitat under the Directive 92/43/EEC (habitat 6220 * “Pseudo-steppe with grasses and annuals of the *Thero-Brachypodietea*”). Some authors report it also inside *Saginetea maritimae* Westhoff, Leeuwen & Adriani 1962 vegetation [135].

The only known population falls within the Special Protection Area (SPA) called “Arcipelago delle Pelagie-area marina e Terrestre” (code ITA040013), which should guarantee its conservation in the medium and long term.

In the Italian Red List, it is reported as Near threatened (NT) (Table 1), showing a high in situ (A) and normal ex situ (NP) priority conservation, and no data about gene pool are available (Table 2 and Table 3).

#### Expected Actions

- Expected Actions *D. scapos*a, almost unknown, is probably relevant in relation to rocket cultivation and should deserve major ex situ efforts to facilitate research;
- in situ conservation to reduce the risk of extinction by increasing the number of individuals of existing wild populations;
- test the unknown chemical composition.

### 4.6. Festuca centroapenninica *(Markgr.-Dann.) Foggi, F. Conti & Pignatti*

On mere taxonomic grounds, *Festuca* L. s.l. represents a problematic critical group of worldwide interest because of its intrinsic high phenotypic variability and nomenclatural complexity [136], and because of its allied genus *Lolium* L. they are among the most widely studied of the non-cereal grasses. The genus *Festuca* s.l. includes two agriculturally important forage crops, the hexaploid *L. arundinaceum* (Schreb.) Darbysh. (syn.: *F. arundinacea* Schreb.) (tall fescue) and the diploid *L. pratense* (Huds.) Darbysh. (Syn.: *F. pratensis* Huds.) (meadow fescue). Other relevant species are *F. rubra* L and *F. ovina* L., as forage and turf. *F.* species are well adapted to abiotic stresses such as heat, drought, and low temperature, but they do not compare well in animal forage provision to *L.* species as *F.* species show poor establishment and comparatively lower quality characteristics [137].

According to some authors [138], it is urgent to elaborate lists based on a taxonomic approach to finding gaps in existing ex situ collections, to assess the conservation status of taxa of ascertained utility to include them in national Red Lists and to identify the most valuable taxa to be included in “preservation mixtures”, as per Commission Directive 2010/60/EU [accessed on 18 November 2021] [139].

In Italy, 83 specific and subspecific taxa of the genus *Festuca* (excluding *Lolium* p.p.) are reported, 17 of which are endemic at the national level [18], 41 are CWRs, of which 10 are endemic (*F. apuanica* Markgr.-Dann., *F. centroapenninica*, *F. gamisansii* Kerguélen subsp. *aethaliae* Signorini and Foggi, *F. imperatrix* Catonica, *F. pignattiorum* Markgr.-Dann., *F. riccerii* Foggi and Gr. Rossi, *F. robustifolia* Markgr.-Dann., *F. veneris* Gr. Rossi, Foggi and Signorini, *F. violacea* Schleich. ex Gaudin subsp. *italica* Foggi, Gr. Rossi and Signorini, *F. violacea* Schleich. ex Gaudin subsp. *puccinellii* (Parl.) Foggi, Gr. Rossi and Signorini) [138]. Due to the high number of endemic taxa and the complexity of the group, the discussion inside this genus will require a specific and separate contribution.

*F. centroapenninica* (syn.: *F. ovina* L. var. *centroapenninica* Markgr.-Dann.) is an endemic taxon of Abruzzo, Lazio, Marche, Umbria, and Tuscany (Central Italy) [18], reported for several sites (e.g. [140,141,142,143])). It is a diagnostic taxon of *Festuco-Koelerietum gracilis* Cortini Pedrotti et al. 1973 association of *Festuco-Brometea* Br.-Bl. et Tx. ex Soó 1947 class, that fits into meso-xerophilous grasslands, with closed and dense herbal coverage, found on a restricted area inside Monti Sibillini National Park [142,144], and falls within priority habitat “Semi-natural dry grasslands and scrubland facies on calcareous substrates (*Festuco-Brometalia*) (* important orchid sites)” (code 6210 *).

This taxon shows a medium in situ (B) and a high ex situ (HP) priority conservation, with no data about chromosome number. In the absence of a domesticated species, the matter of gene pool is not applicable (Table 2 and Table 3).

#### Expected Actions

- in situ conservation, also aimed to a better understanding about the ecology of the species;
- ex situ conservation as there are no accessions in the Italian RIBES seed banks;
- assess its real distribution area and populations size.

### 4.7. Lathyrus apenninus *F. Conti,* L. odoratus *L.*

The genus *Lathyrus* L. belongs to the Fabaceae family and includes around 160 species [145,146]. It is economically important as some species are used in several countries as human food, fodder [147], folk medicine [148], especially for its high protein content [149], and as ornamental crops, for instance, *L. odoratus* [150]. Although the phenolic content of some species of *Lathyrus* is known, including *L. sativus* L. [149], the phytochemical profile and antioxidant capacity of many species of *Lathyrus* still remain unknown. It deserves a special mention *L. cicera* L., one of the oldest cultivated plants in the world, originated in the Mediterranean center of diversity, and together with its close relative, *L. sativus*, is most likely the first crop domesticated in Europe, particularly in the Iberian Peninsula and southern France [151].

In Italy, 38 taxa of *Lathyrus* are reported, 3 of which are endemic to Italy: *L. apenninus*, *L. odoratus*, and *L. jordanii* (Ten.) Ces., Pass. & Gibelli. [18]. *L. jordanii*, reported as DD (Data Deficient) in Molise [29], is not evaluated because it is not reported in the RIBES list but should be studied carefully because there are doubts about the validity of the taxon, which differs from the most widespread *L. niger* (L.) Bernh. only for the stem at the base, provided with cylindrical tuberized roots, mutable character, rarely observed even within the same population attributed to *L. jordanii* (personal observations of E.V. Perrino), and which therefore could be evaluated as an ecotype of *L. niger*.

*L. apenninus* is an endemic of the central Apennines [152], reported in Abruzzo, Marche, Umbria, and Lazio [18], in the woods (especially beech) edges, clearings, and the shrubs near streams [152,153]. Its presence at the edge of the beech forest is probably secondary, where it grows together with *Paeonia officinalis* L. subsp. *italica* N.G. Passal. & Bernardo (other taxon endemic to Central Apennines) [152]. Extant populations of *L. apenninus* and the two closest related species (*L. alpestris* (Waldst. & Kit.) Celak. and *L. vivantii* P. Monts.) live in similar habitat and might be a remnant of a previously larger distributional range that experienced contraction during the Quaternary leading to the present fragmentation [152].

This taxon is reported as Near Threatened (NT) in the Italian National Red List (Table 1) and shows a high in situ (A) and ex situ (HP) priority conservation, with no data about chromosome number and gene pool (Table 2 and Table 3).

*L. odoratus* (Sweet pea) is an important ornamental annual plant of the temperate regions, widespread in gardens and landscapes [154]. It is reported in many Italian regions, but it is inconstant with unclear gaps, evaluated at the regional level as native (Abruzzo, Molise, Campania, Basilicata, Calabria, and Sicily), or alien as naturalized (Tuscany), casual (Piemonte, Lombardia, Trentino-Alto Adige, and Sardinia), and finally also as no longer recorded alien (Lazio and Puglia) [18]. It shows a high in situ (A) and ex situ (HP) priority conservation, with no data about gene pool (Table 2 and Table 3).

#### Expected Actions

- In situ and ex situ conservation, with insights into the geographic limit and size consistency of populations;
- assessment of the endemism status of *L. odoratus*, as it is also reported in other countries;
- study on the distribution at the national level of *L. odoratus*, with clarification of its attribution as native or alien species in the different Italian regions;
- evaluate the status of the crop wild relative of *L. odoratus*, a domesticated species, cultivated as an ornamental plant;
- genetic studies to define the gene pool and the chromosome number of both species;
- phytosociological studies.

### 4.8. Malus crescimannoi *Raimondo*

The genus *Malus* Mill. shows a very complex taxonomy and nomenclature, based on minute morphological characters, often continuous and overlapping, making it arduous to differentiate species and subspecies inside the genus. In fact, some authors attribute to it only 8 species [155], others from 25 to 47 species, depending on the rank assigned to different taxa and on the acceptance of putative hybrids [156], up to 78 species [157]. The differences in classification are mainly due to the taxonomic level at which infrageneric groupings of species are recognized, although there is broad agreement on the species that comprise these groupings [158].

Many taxa of this genus are mentioned on the global priority list for crop wild relatives conservation [159], and on 33 of the crop wild relatives, more than 20 wild species were considered to be a high priority for increased conservation efforts, and all the wild apple species still required further collection [36,160].

*M. komarovii* (Sarg.) Rehder, *M. niedzwetzkyana* Dieck, *M. sieversii* (Ledeb.) M. Roem., and *M. trilobata* (Labill. ex Poir.) C.K. Schneid. are the taxa at major risk, as mentioned on the International Union for Conservation of Nature (IUCN 2021) [https://www.iucnredlist.org, accessed on 1 December 2021] [161].

In Europe, 6 taxa occur, belonging to sect. *Malus* and sect. *Eriolobus* (Ser.) C. K. Schneider [162] or sect. *Florentinae* (Rehder) G. Z. Qian [163], 3 of which, excluding the cultivated *M. domestica* (Borkh.) Borkh., are reported to Italy: *M. crescimannoi*, the only Italian endemism in genus *Malus*, *M. florentina* (Zuccagni) C.K. Schneid. and *M. sylvestris* (L.) Mill. [18].

*M. crescimannoi* belongs to *M.* sect. *Malus* and is differentiated from the two related species, *M. sylvestris* and *M. domestica*, by relevant morphological characters [164]. Two populations are known for this taxon, both in Northern Sicily, from 1000 to 1800 m a.s.l. [164,165]. The oldest of those corresponds with the identification site of the species and is confined to the sub-montane belt of Nebrodi Mts., on siliceous soil (Messina Province) [165], the second on quartzarenitic soils in the neighboring area of Madonie Mts. (Palermo Province) [164]. This tree grows in or at the edge of deciduous woods of *Quercus cerris* L., *Q. petraea* (Matt.) Liebl., and *Fagus sylvatica* L. subsp. *sylvatica* with other Rosaceae such as *Crataegus monogyna* Jacq., *Pyrus ciancioi* P. Marino, Spadaro, G. Castellano & Raimondo, *P. spinosa* L. subsp. *spinosa*, *Pyrus communis* L. subsp. *pyraster* (L.) Ehrh., *Rosa canina* L., and *Sorbus torminalis* (L.) Crantz, and sometimes on the Madonie Mts., also with *M. sylvestris* [164,165]. It flowers in April-May, and fruiting is in October-November [165]. From the phytosociological point of view, this species grows on the Nebrodi Mts. in the *Anemono apenninae–Fagetum sylvaticae* (Gentile 1969) Brullo 1984 *em.* Ubaldi et al. 1987 association, and it is evaluated as a characteristic species of the *Carpino-Fagetea* Jakucs ex Passarge 1968 class [166], ascribable to the “Apennine beech forests with *Taxus* and *Ilex*” priority habitat of Directive 92/43/EEC (code 9210*).

In the Italian Red List, this taxon is reported as Near Threatened (NT) (Table 1), and it shows a high in situ (A) and ex situ (HP) priority conservation (Table 2 and Table 3), and GP2 relative of *M. domestica* (www.cwrdiversity.org/checklist/, accessed on 28 September 2021) [35]. It is noteworthy to mention that the other two wild Italian apples are GP1 (*M. sylvestris*) and GP3 (*M. florentina*) relatives of *M. domestica*, which suggests interesting crossing and breeding initiatives.

#### Expected Actions

- In situ and ex situ conservation need improvement;
- phytosociological studies are needed, especially for the Madonie population(s);
- crossing and breeding with cultivated *M. domestica* and wild *M. sylvestris*.

### 4.9. Phalaris arundinacea *L. subsp.* rotgesii *(Husn.) Kerguélen*

The species of the genus *Phalaris* L. are an important forage, ornamental, birdseed, wetland remediation/restoration, and biofuel crop grown across the globe but are also recognized as invasive wetland species [167,168,169]. The latest taxonomic overviews of *Phalaris*, a complex taxonomic and nomenclatural genus of Poaceae, include 20 taxa [170,171,172,173]. In the Mediterranean region, ten species are reported [174], 9 of which grow in Italy, with only *P. arundinacea* subsp. *rotgesii* is an endemic of the country [18]. At present, this taxon is no longer recorded for Sardinia, the only Italian region where it was reported [18], while it is known in Corsica too [22]. Even if some authors report it for specific localities in Sardinia, such as Sulcis (SW Sardinia) [175] or as an unfavorable species for grazing [52], there is a lack of recent herbarium specimens that testify its current presence in the region.

In the European Red List, the species *Ph. arundinacea* is reported as Least Concern (LC), but no data are available on the threat category of the subspecies *Ph. arundinacea* subsp. *rotgesii* (Table 1). This taxon shows a medium in situ (B) and high ex situ (HP) priority conservation, with no data about gene pool (Table 2 and Table 3).

#### Expected Actions

- Check the existence of the Sardinian population(s) and its geographic distribution inside the region;
- evaluate his aptitude, especially as forage, wetland remediation, and biofuel;
- ex situ conservation can be started involving botanical gardens and seed banks at regional and national levels;
- phytosociological studies, to evaluate the best strategies for its management and conservation;
- genetic studies to define the gene pool.

### 4.10. Vicia brulloi *Sciandr., Giusso, Salmeri & Miniss.,* V. consentina *Spreng.,* V. giacominiana *Segelb.,* V. ochroleuca *Ten. subsp.* ochroleuca, V. tenuifolia *Roth subsp.* elegans *(Guss.) Nyman.*

The genus *Vicia* L. (Fabaceae) includes approximately 190 taxa [176,177] distributed throughout the temperate regions of North and South America, Europe and Asia [178,179], and the Mediterranean Basin is its most important diversity center, while it is still problematic to establish the center of origin [180]. Many species of the genus are useful as food crops and forage, mostly *V. sativa*, which represents one of the most economically important crop species [181,182]. *Vicia* has some critical problems on a taxonomic level, especially for the infrageneric taxa [183] and genetic relationships between crops and their wild relatives, since wild species may serve as a source of new attributes for crop plants [184,185]. Therefore, assessing the genetic variability within the genus is required if the species must be utilized in plant breeding programs. To this purpose, germplasm characterization needs to be performed with several approaches, such as morphology, cytogenetics, biochemistry, and geographic distribution [183].

In Italy, 54 species of *Vicia* are reported, five of which are endemics [18,20]. *V. brulloi*, *V. consentina* and *V. giacominiana* are endemic of a single region, while *V. ochroleuca* subsp. *ochroleuca* and *V. tenuifolia* subsp. *elegans* are widespread in central and southern Italy with some doubts [18]. All taxa show a high (A) in situ priority conservation, except *V. ochroleuca* subsp. *ochroleuca,* which has a medium priority (B), while for ex situ conservation, 4 taxa have high (HP), and only *V. tenuifolia* subsp. *elegans* a normal (NP) priority conservation (Table 2 and Table 3). The gene pool is unknown for all of them (Table 3).

*V. brulloi* has been described from Peloritani (NE of Sicily, Messina municipality), where it grows along the riversides of Torrente Girattimmi near Antillo, on metamorphic outcrops, at 570 m a.s.l., within riparian woods dominated by *Platanus orientalis* L. and *Alnus glutinosa* (L.) Gaertn., in shady conditions and muddy wet soils [20]. It is a member of an endemic plant community referable to *Platano orientalis-Salicetum gussonei* Brullo & Spampinato 1991, falling according to Directive 92/43/EEC within the habitat “*Platanus orientalis* and *Liquidambar orientalis* woods (*Platanion orientalis*)” (code 92C0). It shows significant relationships only with *V. pisiformis* L. and has a chromosome number 2n = 12, with flowering from late February to early May and fruiting from May to July [20]. *V. brulloi* is known only for a circumscript population with no more than 100 individuals within the protected area “Tratto Montano del Bacino della Fiumara di Agrò” (ITA030019) (Special Area of Conservation, SAC)”, and was classified as Critically Endangered (CR) [20].

*V. consentina* is a poorly recorded Calabrian endemism, reported in Sila Massif [186]. The available data on this species are confused because, by mistake, sometimes it has been considered as a synonym of the most widespread *V. pseudocracca* Bertol. [15]. For this reason, the data from the Basilicata region [187,188] and the herbarium specimens from Sicily and Sardinia [115] of *V. consentina* should be referred to *V. pseudocracca*. In the Italian Red List, it is reported as Near threatened (NT) (Table 1).

*V. giacominiana* is a very rare Apulian endemic taxon, reported in a few sites of Salento [189,190,191,192,193,194]. It is linked to environments with human disturbance, especially grazing and fire, phenomena that in any case should be managed in a correct way to avoid negative effects [195,196], providing, for example, a suitable grazing plan for the conservation of itself and its habitat [197]. *V. giacominiana* is found exclusively within annual communities dominated by annual species such as *Stipellula capensis* (Thunb.) Röser & H.R. Hamasha and *Trigonella esculenta* Willd. [191], of *Stipion retortae* O. de Bolòs 1957 alliance, attributed to the annual variant of the priority habitat “Pseudo-steppe with grasses and annuals of the *Thero-Brachypodietea*” (6220 *). In the recent National Red Lists, *V. giacominiana* is considered as Critically Endangered (CR) (Table 1).

*V. ochroleuca* subsp. *ochroleuca* is reported in many places of Tuscany (e.g. [198,199]), Campania (e.g., [200,201]), Basilicata (e.g., [202]), Calabria (e.g., [203,204,205]) and Sicily (e.g., [206,207]), while it is doubtfully occurring in Lazio and Abruzzo [18]. It is a diagnostic taxon of the deciduous woodlands of *Festuco exaltatae-Aceretum neapolitani* Mazzoleni & Ricciardi 1995 association [201], plant community ascribable to the priority habitat “*Tilio-Acerion* forests of slopes, screes and ravines” (code 9180*).

*V. tenuifolia* subsp. *elegans* (syn.: *V. elegans* Guss.) is poorly documented, especially in Lazio and Marche (e.g., [208]). There are some herbarium specimens from Tuscany and Sicily [115,209], while it is no longer recorded in Campania, Basilicata, and Calabria, and finally, it is reported by mistake in Liguria [18]. There are doubts about the endemic character of this taxon because it was observed in other countries, as in Bulgaria at Kalofer [210], and in Azerbaijan [211].

#### Expected Actions

- Genetic studies to define the gene pool, research on ecological traits, vegetation types, and geographic extension of the populations of all *Vicia* taxa;
- neotypification of *V. consentina*, as the original herbarium material, is untraceable [212], and inspection of the herbarium samples attributed to *V. pseudocracca* to verify the correct identification;
- in situ and ex situ conservation strategy of *V. giacominiana*, with the management of pasture and fire, as suggested in the Technical reports of the European Commission [195], and other studies of these topics;
- check the presence of *V. ochroleuca* subsp. *ochroleuca* in Lazio and Abruzzo regions, and of *V. tenuifolia* subsp. *elegans* in Campania, Basilicata and Calabria;
- check the endemic status of *V. tenuifolia* subsp. *elegans*.

## 5. Conclusions

The list of the threatened CWRs endemic to Italy was updated, adding three recently described species. As this work aimed to assess the state of the 29 Italian threatened and endemic CWRs, our research was focused on their geographical distribution, ecology, and in situ and ex situ conservation. Our work, carried out species by species, made it possible to draw up several action plans for improving their conservation and enhancement. Fortunately, at least in our case, enhancement means satisfying material economic and biological needs without additional economic costs, as all the proposed action plans may have material and immaterial feedback. For example, we may cite testing the chemical composition of essential oils for their potential use, improvement of in situ and ex situ conservation involving botanical gardens and seed-banks, and assessing the real distribution area and population size of the species. In some cases, the real endemic status must be checked, too, given that some of the analyzed taxa are reported with doubt also outside Italy. According to the concept of gene pools, this and other feedback are more important for those wild relatives that can exchange genes with their crop relatives, real CWRs. Unfortunately, at the moment, only 6 out of the 29 analyzed CWRs satisfy this concept. In addition, there is a serious lack of data on the ex situ conservation in gene banks, with high priority (HP) in 16 species, while 22 taxa have high priority (A) for in situ conservation. Other gaps are related to the ecology, especially for the plant communities and for 92/43/EEC habitats, with 12 species without syntaxonomic framework. That is why among the many proposed action plans, this work included further research to discover new and/or unknown CWRs.

The provided information can satisfy the needs expressed by the EU-white papers on the future science for European policies, given that this information can be used by policymakers and institutions to move from potentiality to strategy of biological conservation. In this light, the proposed recommendations and action plans would be useful and applicable not only in Italy but also in other European and extra-European countries.

All these statements and suggested actions have the ultimate goal to preserve the biological nature and the genetic features of the Italian endemic threatened Crop Wild Relatives, which, due to their restricted geographical distribution, risk becoming extinct in a short time.

## Figures and Tables

**Table 1:** List of Italian threatened endemic crop wild relatives, reasons of threatening.

Taxa	Endemic to	IT	IS	1	2	3	4	5	6	7	8	9	10	11
*Arrhenatherum elatius* subsp. *nebrodense*	Sicily	x		LC						NT				
*Barbarea rupicola*	Sardinia	x		LC							LC			
*Brassica baldensis*	Veneto	x	x							VU				
*Brassica glabrescens*	Friuli-Venezia Giulia	x	x	VU			VU^F^	VU	NT	NT			II	x
*Brassica macrocarpa*	Sicily	x	x	CR	CR	CR		EN	CR	CR			II *	x
*Brassica rupestris* subsp. *hispida*	Sicily	x	x	NT *	EN	EN				VU				
*Brassica rupestris* subsp. *rupestris*	Calabria, Sicily	x	x	NT *	LR	LR				LC				
*Brassica tardarae* **	Sicily	x	x									VU †		
Brassica trichocarpa **	Sicily	x	x							NT		CR ‡		
*Brassica tyrrhena*	Sardinia	x	x							LC				
*Brassica villosa* subsp. *bivonana*	Siciliy	x	x	NT *	LR	LR				LC				
*Brassica villosa* subsp. *brevisiliqua*	Siciliy	x	x	NT *						NT				
*Brassica villosa* subsp. *drepanensis*	Siciliy	x	x	NT *	LR	VU				VU				
*Brassica villosa* subsp. *tineoi*	Siciliy	x	x	NT *	VU	VU				LC				
*Brassica villosa* subsp. *villosa*	Siciliy	x	x	NT *	CR	CR				LC				
*Daucus broteroi*	Peninsula	x	x	DD						LC				
*Daucus carota* subsp. *rupestris*	Sicily	x	x	LC *	EN	EN				EN				
*Daucus nebrodensis*	Sicily	x	x							LC				
*Diplotaxis scaposa*	Sicily	x	x							NT				
*Festuca centroapenninica*	Appennine	x								LC				
*Lathyrus apenninus*	Appennine	x	x							NT				
*Lathyrus odoratus*	Peninsula, Sicily	x	x	NT			CR ^M^ LR ^A,L^			LC				
*Malus crescimannoi*	Sicily	x	x	DD						NT				
*Phalaroides arundinacea* subsp. *rotgesii*	Sardinia	x		LC*										
*Vicia brulloi* **	Sicily	x	x									CR §		
*Vicia consentina*	Calabria	x	x							NT				
*Vicia giacominiana*	Apulia	x	x		CR		CR ^P^	VU	CR °	CR				
*Vicia ochroleuca* subsp. *ochroleuca*	Peninsula, Sicily	x	x							LC				
*Vicia tenuifolia* subsp. *elegans*	Peninsula, Sicily	x	x				VU ^L^			NT				
Adapted and updated from Landucci et al. [1], Magrini et al. [14], and Perrino and Perrino [6].														
** Not reported in previous works [1,6,14].														
**Endemic to** = as reported in “An updated checklist of the Vascular flora native to Italy” [18].														
**IT = ITPGRFA Annex I**: Taxa included in Annex I of the International Treaty on Plant Genetic Resources for Food and Agriculture (ITPGRFA) [17].														
**IS = ISTAT**: Taxa mentioned by the Italian Institute of Statistics (ISTAT) for cultivated areas and yield between 2019 and 2021 (ISTAT) [26].														
1: **BILZ ET AL. 2011**: Taxa included in the European Red List [27]: DD = Data deficient, LC = Least concern, NT = Near Threatened, VU = Vulnerable, CR = Critically endangered. * The category refers only to the species because Bilz et al. [27] do not report the subspecies.														
2–4: **CONTI ET AL. 1997**: Taxa included in the Italian Regional Red Lists [29]; 2 = Italy, 3 = Sicily, 4: A = Abruzzo, F = Friuli-Venezia Giulia, L = Lazio, M = Molise, P = Apulia, CR = Critically endangered, EN = Endangered, VU = Vulnerable, LR = Lower risk.														
5: **CONTI ET AL. 1992**: Taxa included in the Italian National Red Book [28]: EN = Endangered, VU = Vulnerable.														
6: **POLICY SPECIES**: Taxa included in the Italian Red List of Policy Species [30]: CR = Critically endangered, NT = Near Threatened. ° Non-policy species.														
7: **ORSENIGO ET AL. 2018**: Taxa included in the new Red List of the Italian endemic flora [31]: CR = Critically endangered, EN = Endangered, VU = Vulnerable, NT = Near Threatened, LC = Least concern.														
8: **ORSENIGO ET AL. 2021**: Taxa included in the new Italian National Red List [32]: LC = Least concern.														
9: **OTHER IUCN CARDS** = Category of risk reported in other specifics works: † = [21], ‡ = [19], § = [20]; VU = Vulnerable, CR= Critically endangered.														
10: **EUROPEAN COMMISSION (1995–2007)** = Annex II of the Directive 92/43/EEC; (*) = priority species [34].														
11: **BERN CONVENTION (Council of Europe, 1979)** = Appendix I of Bern Convention [33].														

**Table 2:** Italian threatened endemic crop wild relatives, their status of ex situ and in situ conservation, and relationships with plant communities and/or habitat of the Directive 92/43/EEC.

Taxa	Ex Situ Priority			In Situ Priority		*Syntaxon* and/or Habitat 92/43 EEC (* Priority)
	HP	NP	ZP	A	B	
*Arrhenatherum elatius* subsp. *nebrodense*	x			x		*Arrhenathero nebrodensis-Quercetum cerridis*, *Linarion purpureae* (8130)
*Barbarea rupicola*		x			x	?
*Brassica baldensis*	x			x		(8210)
*Brassica glabrescens*		x		x		*Centaureo dichroanthae-Globularietum cordifoliae* (62A0)
*Brassica macrocarpa*			x	x		*Scabioso-Centaureetum ucriae* subass. *brassicetosum macrocarpae* (8210), *Euphorbietum dendroidis* (5330)
*Brassica rupestris* subsp. *hispida*			x	x		?
*Brassica rupestris* subsp. *rupestris*			x	x		*Diantho rupicolae-Helichrysetum panormitani*, *Scabioso creticae-Centauretum ucriae* (8210)
Brassica tardarae **	x			x		*Brassico rupestris-Centauretum saccensis* (8210)
Brassica trichocarpa **	x			x		(5330)
*Brassica tyrrhena*		x			x	*Helichryso saxatili-Cephalarietum mediterraneae* (8210)
*Brassica villosa* subsp. *bivonana*			x	x		?
*Brassica villosa* subsp. *brevisiliqua*			x	x		?
*Brassica villosa* subsp. *drepanensis*			x	x		*Scabioso-Centauretum ucriae* subass. *typicum* (8210)
*Brassica villosa* subsp. *tineoi*			x	x		*Brassico tinei-Diplotaxietum crassifoliae* (8210)
*Brassica villosa* subsp. *villosa*			x	x		?
*Daucus broteroi*	x				x	?
*Daucus carota* subsp. *rupestris*	x			x		*Erico multiflorae-Coronilletum glaucae* (5410)
*Daucus nebrodensis*	x				x	?
*Diplotaxis scaposa*		x		x		*Filagini-Daucetum lopadusani* (6220 *)
*Festuca centroapenninica*	x				x	*Festuco-Koelerietum gracilis* (6210 *)
*Lathyrus apenninus*	x			x		?
*Lathyrus odoratus*	x			x		?
*Malus crescimannoi*	x			x		*Carpino-Fagetea* (9210 *)
*Phalaroides arundinacea* subsp. *rotgesii*	x				x	?
Vicia brulloi **	x			x		*Platano orientalis-Salicetum gussonei* (92C0)
*Vicia consentina*	x			x		?
*Vicia giacominiana*	x			x		*Stipion retortae* (6220 *)
*Vicia ochroleuca* subsp. *ochroleuca*	x				x	*Festuco exaltatae-Aceretum neapolitani* (9180 *)
*Vicia tenuifolia* subsp. *elegans*		x		x		?
TOTAL	16	5	8	22	7	
** Not reported in previous works [1,6,14].						
**Ex situ priority conservation**. **HP**: Taxa with high priority (zero accessions); **NP**: Taxa with normal priority (1–4 accessions); **ZP**: Taxa with no priority (5–140 accessions).						
**In situ priority conservation**. **A**: Includes native taxa related to a crop of worldwide and national importance for food and agriculture, which are included in (at least) one of the following sources: IUCN European Red List [27], Regional Red List (national catalog) [29], National Red Lists [28,30,31,32], Other IUCN cards [19,20,21], Annex II of the Directive 92/43/EEC [34], Appendix I of Bern Convention [33]. These taxa need specific protection and/or monitoring measures. **B**: Includes native taxa related to important crops, which are not included or are reported as Least concern (LC) or as Data deficient (DD) in the lists mentioned above. These taxa need specific protection and/or monitoring measures.						
**Habitat 92/43 EEC and/or Vegetation type (Italy)**. Vegetation type (see reference in the text when discussing the species).						

**Table 3:** Italian threatened endemic crop wild relatives, their status of ex situ and in situ conservation, and relationships with gene pool (GP).

Taxa	Gene Pools (GP)			Ex Situ Priority			In Situ Priority	
	GP1	GP2	GP3	HP	NP	ZP	A	B
*Arrhenatherum elatius* subsp. *nebrodense*				x			x	
*Barbarea rupicola*					x			x
*Brassica baldensis*				x			x	
*Brassica glabrescens*					x		x	
*Brassica macrocarpa* Guss.		x	x			x	x	
*Brassica rupestris* subsp. *hispida*						x	x	
*Brassica rupestris* subsp. *rupestris*		x				x	x	
Brassica tardarae **				x			x	
Brassica trichocarpa **				x			x	
*Brassica tyrrhena*					x			x
*Brassica villosa* subsp. *bivonana*						x	x	
*Brassica villosa* subsp. *brevisiliqua*						x	x	
*Brassica villosa* subsp. *drepanensis*		x				x	x	
*Brassica villosa* subsp. *tineoi*						x	x	
*Brassica villosa* subsp. *villosa*		x				x	x	
*Daucus broteroi*				x				x
*Daucus carota* subsp. *rupestris*	x			x			x	
*Daucus nebrodensis*				x				x
*Diplotaxis scaposa*					x		x	
*Festuca centroapenninica*				x				x
*Lathyrus apenninus*				x			x	
*Lathyrus odoratus*				x			x	
*Malus crescimannoi*		x		x			x	
*Phalaroides arundinacea* subsp. *rotgesii*				x				x
Vicia brulloi **				x			x	
*Vicia consentina*				x			x	
*Vicia giacominiana*				x			x	
*Vicia ochroleuca* subsp. *ochroleuca*				x				x
*Vicia tenuifolia* subsp. *elegans*					x		x	
TOTAL	1	5	1	16	5	8	22	7
** Not reported in previous works [1,6,14].								
**Ex situ priority conservation**. **HP**: Taxa with high priority (zero accessions); **NP**: Taxa with normal priority (1–4 accessions); **ZP**: Taxa with no priority (5–140 accessions).								
**In situ priority conservation**. **A**: Includes native taxa related to a crop of worldwide and national importance for food and agriculture, which are included in (at least) one of the following sources: IUCN European Red List [27], Regional Red List (national catalog) [29], National Red Lists [28,30,31,32], Other IUCN cards [19,20,21], Annex II of the Directive 92/43/EEC [34], Appendix I of Bern Convention [33]. These taxa need specific protection and/or monitoring measures. **B**: Includes native taxa related to important crops, which are not included or are reported as Least concern (LC) or as Data deficient (DD) in the lists mentioned above. These taxa need specific protection and/or monitoring measures.								

## Data Availability

Not applicable.
